# Decolonizing climate change response: African indigenous knowledge and sustainable development

**DOI:** 10.3389/fsoc.2024.1456871

**Published:** 2024-11-20

**Authors:** James Ojochenemi David

**Affiliations:** Department of Police Practice, University of South Africa, Pretoria, South Africa

**Keywords:** anthropogenic global warming, scientism, African indigenous knowledge, decoloniality, education for sustainable development (ESD), exemplary ethical communities (EEC)

## Abstract

**Background:**

Anthropogenic Global Warming (AGW) poses a critical challenge necessitating effective global climate change mitigation efforts. However, the pervasive influence of scientism in AGW discourse often marginalizes Indigenous perspectives crucial for addressing climate impacts, particularly in Africa where adaptive capacity is limited.

**Objective:**

This study, rooted in Transformative Learning Theory and Ubuntu philosophy, employs critical qualitative research methods to examine how scientism shapes AGW discourse epistemologically and ethically. It explores the hindrances posed by climate change denialism and ecomodernism due to scientism while advocating the integration of African Indigenous Knowledge Systems (AIKs) into climate response strategies, particularly within the African education landscape.

**Methods:**

Drawing on the theoretical frameworks of Transformative Learning and Ubuntu philosophy, and informed by critical qualitative research methodology, this research analyzes the role of scientism in AGW discourse. It investigates its implications for Education for Sustainable Development (ESD) and discusses arguments for the inclusion of AIK in educational and policy frameworks.

**Results:**

The study reveals that scientism perpetuates epistemological biases that undervalue AIK, thereby impeding comprehensive climate response strategies. Pathways are proposed that promote AIK integration and mainstreaming, thereby decolonizing climate response efforts and enhancing ESD within Africa’s educational institutions.

**Conclusion:**

Integrating insights from AIK, construed in terms of ‘exemplary ethical communities’ (EEC), into climate change responses is pivotal for fostering inclusive and effective strategies. This approach not only addresses the ethical imperatives of decolonization but also enhances resilience and sustainability in climate-vulnerable regions.

**Significance:**

This study contributes to scholarship by highlighting the urgent need to diversify climate response strategies through the inclusion of AIK. By advocating for the integration of wisdom from EECs, it advances discussions on decoloniality within climate change discourse, emphasizing the importance of Indigenous knowledge in global sustainability efforts.

## 1 Introduction

The debate surrounding Anthropogenic Global Warming (AGW) reflects both scientific consensus and ideological resistance. AGW refers to the human-induced increase in greenhouse gases like carbon dioxide (CO₂) that accelerates the greenhouse effect, driving climate change (Okoliko, 2018). At the heart of this debate is scientism—the belief that science is the most authoritative form of knowledge for policymaking (Blue, 2018). While science strongly supports the need for urgent climate action (Hamilton, 2016), two influential perspectives—denialism and ecomodernism—seek to maintain business-as-usual practices through technological solutions (Ruser and Machin, 2016). Climate change denialists argue that the connection between human activities and global warming if it exists at all, is minimal. They point to the lack of unanimity among scientists (Milfont et al., 2021; Parks, 2020). Disputing the conclusions of the Intergovernmental Panel on Climate Change (IPCC), deniers contend that AGW proponents’ assertions lack merit (Parks, 2020). Some do this by invoking the influence of solar irradiance and atmosphere–ocean interactions, both of which, in their view, have warming effects at least as significant as those attributed to CO2-driven mechanisms, all based on well-established scientific principles (Idso et al., 2015). On the other hand, ecomodernism acknowledges climate change but advocates for the use of technology and innovation to decouple human well-being from environmental impacts, suggesting that modernity and economic growth can coexist with ecological sustainability. Unlike traditional environmentalism, ecomodernists emphasize the potential of technological solutions, such as renewable energy and nuclear power, or geoengineering to address climate change (Hällmark, 2023; Adelman, 2017). Although with different emphasis, the ecomodernism perspective, like denialism, tends to promote the business-as-usual capitalist framework, rather than reducing consumption and altering societal structures to achieve sustainability. Thus, as Norgaard (2019: 439) has observed, “Emotions of fear and cultures of cognition regarding the end of the modern capitalist system or ‘life as we know it’ have generated climate denial of the far right and a combination of green capitalist technological fixes and climate apathy on the left.”

Both camps, despite their differences, rely on a belief that technology alone can solve climate challenges without addressing the deeper structural changes required in societal values, ethics, and power relationships (Hällmark, 2023; Escobar, 2015). This technocentric focus reflects a legacy of colonial and epistemic domination of the West over the rest, which continues to shape climate policies and global power dynamics (Okoliko and David, 2021; Kreeft, 2007), paying little attention to the limits of technology (Ruser and Machin, 2016). The colonial legacy persists in how certain forms of knowledge are privileged over others, sidelining Indigenous epistemologies and their potential to contribute meaningfully to sustainable development (Seehawer, 2018). By avoiding critical conversations about who controls knowledge, whose knowledge matters, and how responsibility is distributed globally, these approaches perpetuate inequalities (Manthalu and Waghid, 2019). Industrialized nations, such as the U.S. and China, dominate climate policy agendas while poorer regions like Africa—despite contributing the least to global emissions—suffer disproportionately from climate impacts (Hill et al., 2020; Okoliko and David, 2021).

In Africa, climate change manifests through erratic rainfall, droughts, floods, and rising health challenges (Camfield et al., 2020; Zhai et al., 2021). These impacts threaten food security and livelihoods, exposing the region’s low adaptive capacity, which has been shaped in part by the marginalization of Indigenous Knowledge Systems (IKS) through colonial education structures (Mandikonza, 2019). Despite growing calls to integrate African Indigenous Knowledge (AIK) with Western science (Apraku et al., 2018; McKinley, 2019), efforts often focus on extracting ‘useful’ elements of Indigenous knowledge, reinforcing a neocolonial dynamic that undermines epistemic diversity (Thompson et al., 2020).

The Education for Sustainable Development (ESD) agenda promoted by UNESCO (2020a) recognizes the importance of diverse knowledge systems, but the challenge lies in creating genuine synergies between Indigenous and Western epistemologies without assimilating or subordinating one to the other. Scholars argue for climate education and research that honour indigenous methodologies, worldviews, and practices (Shava, 2013) to disrupt the colonial legacy embedded in formal education. This shift is essential for improving Africa’s resilience and advancing global climate action that is equitable, inclusive, and just. Accordingly, this study argues that overcoming the limitations of denialist and ecomodernist approaches requires a shift away from purely technological fixes toward ethically grounded, epistemically diverse climate solutions. Addressing climate change effectively demands an acknowledgment of the colonial legacies in knowledge systems and a commitment to equity and justice in climate governance—ensuring that local, context-specific knowledge systems play a vital role in adaptation and mitigation efforts.

Hence, this study critically engages the scientism that often shapes climate science to demonstrate how the former undermines the contribution of AIK for optimal outcomes in climate change response. It highlights the inherent epistemological flaws and the resultant ethical implication of the political inaction that scientism promotes, by contributing to the scholarly views on the danger of over-relying on technical arguments vis-à-vis the scientific facts on climate change, which risks undermining the promptness and efficiency of response (Beale, 2019; Okoliko, 2018). It will here be argued that this scientistic approach is exclusionary of other epistemic traditions and essentially undermines education for sustainable development (ESD) in Africa. An AIK-informed response embedded in a holistic appreciation of the human-environment relationship is shaped by an eco-reverence (Van Norren, 2022; van Norren, 2020; Ramose, 2015). This approach allows for the acknowledgement of uncertainty and a deep appreciation of the inherent limits of human knowledge. It paves the way for essential attitudinal shifts, which, in turn, necessitate a paradigmatic transformation in the epistemological approach to sustainability education within African higher education institutions. Such a shift can therefore be instrumental in mitigating the adverse impacts of scientism in climate change response, ultimately contributing to more effective policy resolutions within the broader context of the pursuit of ESD.

The following section offers a concise review of global warming, the ongoing debates surrounding AGW and its consequences, emphasizing the challenges presented by scientism. In the subsequent part, the methodological approach, and the underlying frameworks, notably the Transformative Learning Theory and the Ubuntu paradigm, are elucidated to foreground their relevance to climate action and ESD. Finally, the last section delves into the applicability of an AIK-informed approach in comprehending an Afro-centric transformation of the AGW debate. This in-depth exploration provides the foundation for engaging with UNESCO’s recommendation that ESD incorporate “indigenous knowledge and practices” (UNESCO, 2020a, p. 20), specifically from the perspective of African Indigenous Knowledge (AIK) and more particularly, the Ubuntu philosophy.

## 2 Making sense of the anthropogenic global warming debate

Global warming is defined as an increase in global mean surface temperature (GMST) over a 30-year period, expressed relative to pre-industrial levels (IPCC, 2019). Light energy from the sun is partly transformed in the Earth’s atmosphere into infrared radiation, which is absorbed and re-emitted by greenhouse gases. This greenhouse effect plays a crucial role in maintaining life on Earth by regulating surface temperatures (Okoliko, 2018). However, the introduction of greater quantities of greenhouse gases—such as carbon dioxide (CO₂), methane (CH₄), and nitrous oxide (N₂O)—from human activities has disrupted this balance, causing a steady rise in global temperatures around which scholarship on climate change revolves (Letcher, 2021).

Technological progress, from the so-called First Industrial Revolution to the current Fourth Industrial Revolution (4IR), has transformed global economies, and improved production efficiency, enhancing the quality of life. These advances have, however, come at a steep environmental cost. Recent studies show that human activities caused 0.9 to 1.3°C of warming between 1850–1900 and 2010–2019 (Gillett et al., 2021). Crucially, two-thirds of the 1°C warming over the past century occurred after 1975, driven by the rapid exploitation of natural resources (UNESCO, 2020b). The IPCC (2018) warns that the effects of past and current anthropogenic emissions will persist for centuries, causing long-term impacts such as sea-level rise and ecosystem changes, with warming of 1.5°C expected as early as 2040. According to the IPCC (2023) report, the global surface temperature has risen by about 1.09°C since the late 19th century, primarily due to human activities such as fossil fuel combustion and deforestation. This increase in temperature is linked to more frequent and intense heatwaves, droughts, and heavy precipitation events, which disrupt ecosystems and human systems. Projections indicate a significant likelihood of surpassing the 1.5°C threshold within the next few decades, or sooner, leading to severe and potentially irreversible impacts on climate systems and biodiversity. Additionally, the annual global extraction of both renewable and non-renewable resources has doubled since 1980 (UNESCO, 2020b).

The modern discourse on climate change is shaped by a long history of environmental awareness and calls for sustainable development. The 1987 Brundtland Report emphasized that global resources are limited, and unsustainable development must be curbed. This led to Agenda 21, a call for action that essentially highlights the following key ideas, namely: (a) Developing countries cannot pursue the same growth model as industrialized nations without further environmental degradation; (b) Unsustainable consumption and production patterns need to change to safeguard the environment; (c) Developed nations must bear most of the cost of climate mitigation due to their historical responsibility; (d) Despite challenges, sustainable development is achievable if strategies are implemented globally (UNCED, 1992). Education for Sustainable Development (ESD) arose from these calls for action to promote sustainable practices, knowledge, and ethics.

In response to these initiatives, the fossil fuel industry launched a counter-narrative that cast doubt on climate science and created confusion around the urgency of action (Brulle, 2023; Beder, 2014). This deliberate disinformation campaign continues to shape political debates and stall climate action, particularly in nations reliant on fossil fuel economies. In this context, Norgaard (2019) has argued that climate change denialists often view climate science as part of a political strategy rather than an objective pursuit of knowledge, believing that the data is influenced by the motives of scientists and institutions they distrust. They reject climate studies based on the perceived social-political dynamics that generate them, seeing climate science as a threat to their values and way of life, resulting in a dismissal of the urgency of climate change.

Another response to the 1992 UNCED call for action has been ecomodernist solutions and the technological optimism that dominate many mainstream climate strategies, emphasizing “techno-fixes” like geoengineering and automation to address climate challenges (Ruser and Machin, 2016; Adelman, 2017). This approach unlike the denialist acknowledges climate change and seeks sustainability through technological solutions. However, as Hällmark (2023) has pointed out, the ecomodernist responses, despite being initially critical of the nature-society dualism that perpetuates ecological degradation, display a tendency to depoliticize environmental issues by presenting technology as a neutral solution. Indeed, as highlighted by Blue (2018, p. 546) “With roots in statistical modelling, dominant techno-scientific representations of climate change facilitate technical solutions that can displace important social, cultural and ethical considerations.” Such approaches essentially diminish the significance of public engagement and accountability in climate politics. Fundamentally, they separate nature from society, overlooking the complex interdependencies between human actions and environmental impacts, leading to a simplistic understanding of sustainability (Hällmark, 2023). Furthermore, by emphasizing technological progress without addressing the underlying socio-economic structures, ecomodernism risks perpetuating existing inequalities and environmental degradation, rather than fostering genuine ecological responsibility and transformative change. Hence, both denialism and ecomodernist approaches have been criticized for promoting business-as-usual practices without addressing deeper issues of ethics, values, and systemic inequalities (Escobar, 2015; Hällmark, 2023).

The emphasis on technological solutions mirrors a colonial legacy, where knowledge systems from the Global South, including Indigenous ecological knowledge, are marginalized or co-opted into Western frameworks (Claar, 2022). This epistemic inequality not only limits climate resilience but also perpetuates dependency on industrialized nations. Efforts to limit global warming through fossil fuel reduction are not just scientific imperatives but also reflect moral and political responsibilities. As also underlined in Agenda 21 (UNCED, 1992), countries in the Global North bear the brunt of responsibility for emissions, and climate justice therefore requires that they support vulnerable regions like Africa in building adaptive capacities (Hill et al., 2020; Beder, 2014). The climate crisis cannot be effectively addressed without acknowledging and dismantling the unequal global power structures that shape access to resources and influence policymaking (IPCC, 2023; Kemp et al., 2022). It will here be argued that achieving this shift in global power structures requires paying close attention to the influences of scientism in climate change action, especially on the side of denialism.

While proponents of AGW emphasize the significant impact of human activities, with various studies also confirming an overwhelming scientific consensus (Powell, 2017; IPCC, 2018), public awareness and understanding of this consensus remain limited, leading to two influential attitudes: outright denial or downplaying the significance of human activities in global warming (Hamilton, 2016; Parks, 2020). In the US, an estimated 15% do not believe in climate change (Gounaridis and Newell, 2024). These individuals often point to the complexities and nuances in climate science as a basis for their scepticism. Some climate change deniers go as far as labelling the entire global warming theory as a “cruel hoax” that will result in deprivation and starvation for many (Fowler, 2012). Others argue that the best policy response should be determined solely by objective and indisputable scientific facts (Van der Sluijs et al., 2010). Until such ‘irrefutable scientific’ evidence is presented, these sceptics are unwilling to consider climate policy responses, particularly concerning carbon emission reductions (Fischer, 2019). This stance among climate change deniers is rooted in what could be characterized as scientism (Blue, 2018; Mizrahi, 2017), while at the same time using the uncertainties inherent in scientific investigation as a pretext to delay climate action.

Although climate denialism has heavily influenced policy over the past decades and hindered meaningful action, these attitudes are not a global phenomenon. Evidence suggests substantial public support for climate initiatives. For example, a comprehensive global survey conducted through the Gallup World Poll, involving 129,902 participants aged 15 and above across 125 countries, explored financial willingness and perceptions surrounding climate action. The survey revealed that 69% of respondents are willing to allocate 1% of their monthly household income to combat global warming, reflecting widespread readiness to support mitigation efforts. Furthermore, 86% endorsed pro-climate social norms, highlighting a strong collective commitment to environmental responsibility and signalling that public sentiment is aligned with urgent global climate goals (Andre et al., 2024). This is indeed encouraging.

Nevertheless, climate denialists should not be ignored because their arguments often reflect deeper socio-cultural concerns and mistrust of established institutions, which can provide insights into public sentiment and political dynamics. Engaging with their perspectives can also foster a more comprehensive understanding of the societal implications of climate science and the political strategies surrounding it. Moreover, addressing their concerns may help bridge divides and promote more constructive global dialogue on climate change (Norgaard, 2019). In particular, it is important to understand the scientism inherent in denialist climate science. For the denialist, “science is viewed as the sole legitimate means of attaining true knowledge”, even by those who aren’t necessarily scientists themselves (Mizrahi, 2017; Blue, 2018), while at the same time downplaying the cultural norms and economic interests that underpin their arguments. Also, scientism often links reality exclusively to tangible matter and sensory experiences, seemingly disregarding the inherent limitations of human knowledge and the effectiveness of other valid forms of understanding, including philosophical, religious, poetic, and artistic modes of knowledge (Okoliko, 2018).

This science-policy interface vis-à-vis climate change uses arguments that primarily hinge on the (over) emphasis on science-based objectivity and certainty (Miller, 2012). By overemphasizing the “deep disagreement among scientists on scientific issues that must be resolved before the man-made (*sic*) global warming hypothesis can be validated,” contrarians have over the past decades argued that policy response must be only based on the attainment of scientific consensus and certainty regarding AGW (Idso et al., 2015, p. xi). This approach aligns with the “predictive paradigm, “which encompasses both epistemological and ethical dimensions (Okoliko, 2018; Charlesworth and Okereke, 2010). The underlying assumptions of this model encompass the need to eliminate “uncertainty” as a prerequisite for effective policy, coupled with faith in human capacity, especially that of “experts”, to accomplish this objective (Van der Sluijs et al., 2010). This epistemological and ideological stance also rests on the belief that humans should extract maximum material benefit from the Earth System within legal and ecological limits, and the reliance on economic cost–benefit analysis to underpin this principle (Okoliko, 2018). Paradoxically, this scientism-based approach, which can be viewed as an “unscientific belief,” (Sutter, 2015) has served as a constraint on progress in climate change policy globally, due to the disproportionate influence of major geopolitical and economic interest groups.

### 2.1 Limitation in scientism for AGW

The concept of Anthropogenic Global Warming (AGW) is closely intertwined with a prevailing attitude of ‘development-at-all-costs’ driven by technology. Climate deniers often downplay the negative environmental consequences of this reality, subscribing to the “wait-and-see” implications of scientism (Okoliko, 2018). As previously discussed, the industrialized society maintains a predominant human perspective towards the Earth, marked by a subject-object relationship, largely disregarding the evident harmful consequences (Charlesworth and Okereke, 2010). This perspective mirrors the scientific-mechanistic worldview of the early Modern Era (1500–1800), which laid the foundation for the industrial revolution. Thinkers like Descartes, Newton, and Bacon played a role in promoting this anthropocentric mindset (Capra, 1982). During this age of reason, technical knowledge often overshadowed practical wisdom. Even in contemporary times, knowledge that leads to invention is often highly prized over wisdom. However, the conquest of nature through technology seemed to have led to the conquest of people, who employed nature as a tool (Kreeft, 2007).

This techno-progress discourse, influenced by the powerful forces of globalization, has also made inroads into traditional societies, including those in Sub-Saharan Africa. Consequently, the profound reverence for nature that underlies the traditional worldviews of many African communities is gradually giving way to a modernist perspective that sees nature primarily as a resource to be exploited to human ends (Ruffin et al., 2016). As Goulet (1997, p. 1163) aptly questioned: “Should nature be viewed solely as raw material for Promethean exploitation by humans, or as the larger womb of life in which humans exist, move, and have their being, and whose rhythms and laws they must respect?.” Addressing these ethical concerns requires challenging several of the assumptions that underlie Western approaches to climate change issues, including the stance that advocates for greater certainty of AGW knowledge before practical policy responses. In this regard, Miller (2012, p. 220) has pointed to some of the weaknesses underpinning how science has been used in debates on AGW:

(1) an emphasis on “facts” and demand for “proof”; (2) a view of theories that equates them with unsubstantiated guesses; (3) a strong discomfort with uncertainty and unresolved questions; (4) a failure to recognise the importance of scale and context in recognizing trends and formulating explanations; and (5) a rejection of scientific consensus because it is perceived as politically or philosophically motivated.

However, not only denialist discourses but several aspects of the prevalent linear model that governs the interface between science and policy are also affected by similar conceptions, leaving little room for embracing uncertainty. Approaches that assume that uncertainty must be eliminated before taking any action overlook the fundamental limits of human knowledge and the nature of science itself (Okoliko, 2018). Furthermore, such approaches oversimplify the intricacies of the Earth System. Indeed, while the empirical approach to science has been instrumental in advancing technological development, the relentless pursuit of scientific consensus has been shown to impede policy responses to global warming (Chinn et al., 2018; Okoliko, 2018). Ultimately, such approaches thus neglect the reality that human knowledge is not entirely amenable to the strained notions of absolute objectivity and certainty (Mehta et al., 2001), downplaying the complexities and unpredictability of Earth systems and failing to acknowledge the vested interests, cultural bias, and diverging values that underpin any knowledge system.

The ethical implications are equally significant. The intensified pursuit of certainty through increased research not only hinders the formulation of effective policy responses but also perpetuates or exacerbates the activities responsible for climate change. Many growth-oriented economies, driven by the pursuit of economic expansion, tend to overlook the detrimental global consequences of their actions (Petersen et al., 2019; Okoliko, 2018). In this regard, the concept of the Capitalocene, introduced by Moore (2013), explains the concentration of anthropogenic agency in modern growth-based market economies that have intensified resource extraction and consumption, often ‘externalising the costs onto nonhuman species and environments’ (Brightman and Lewis, 2017). It is within this framework that climate change and environmental degradation have accelerated, thereby necessitating a re-evaluation human-environment relationship (Bold, 2019). The ethical concern here revolves around determining the moral permissibility of human actions.

The ‘scientistic’ worldview that prioritizes utility over morality thus necessitates an ethical re-examination, especially when considering climate change education in the African context. Specifically, within Africa’s educational institutions, sufficient epistemic democracy is yet to fully take place due to the historical marginalization of indigenous knowledge (Smith et al., 2018; Lotz-Sisitka et al., 2017). This marginalization has largely been attributed to the dissemination of scientific racism and the promotion of cultural homogenization during colonial expansion (Manthalu and Waghid, 2019). As Blue (2018) argues concerning scientism, “a normative stance that grants implicit authority to scientific and technical experts to define the meaning of public issues, limits the democratic potential of such efforts.” Unless this flawed approach to climate change science is rectified, the world faces additional preventable climate change risks, disproportionately affecting the most vulnerable populations, regardless of their minimal contribution to the problem. As the existing scientific records indicate, global warming, while influenced by some natural factors, is fundamentally driven by human activities (Milfont et al., 2021). To catalyze necessary climate action, the plurality of worldviews and issues of global justice cannot continue to be disregarded.

Hence, among the numerous arenas where change is called for, is the pressing need for transformation in the realm of Education for Sustainable Development (ESD). As Barma et al. (2015) have highlighted, the inherent uncertainties within climate science pose challenges for the comprehensive inclusion of climate change in formal science education. They propose a need for pedagogical, epistemological, and cultural shifts to better address the essence and breadth of climate science. This proposition underscores a recognition of the risks associated with imposing only a Western epistemological framework on other knowledge paradigms, which is a tendency observed even in formal educational institutions in Africa (Heleta, 2016).

## 3 Methodology and theoretical framework

In pursuit of the overarching research objective, which aims to provide insights into the ongoing academic and policy discussions regarding the intersections of AGW and ESD, this study embraces a Critical Qualitative Research Method (CQRM). This approach amalgamates critical theory with qualitative research methods and finds common use in the social sciences and humanities, facilitating the examination of social phenomena, power dynamics, and the foundational ideologies that shape human experiences (Cannella and Lincoln, 2017). CQRM seeks to address fundamental questions, including the portrayal of specific groups within various discourses, practices, and societal structures, the scrutiny of suppressed or erased forms of knowledge within research contexts, the incorporation of narratives and perspectives from research communities into the knowledge generation process, the identification of instances where oppression and exclusion might be concealed as equity within various discourses, and the examination of how elite groups from diverse social backgrounds define values, constructs, and rhetoric, thereby reinforcing existing power structures (Cannella and Lincoln, 2017). The CQRM is, among other things, especially oriented towards social justice and societal transformation (Cannella and Lincoln, 2017). Accordingly, this methodological stance is suitable for critically engaging with the ethical, epistemic, and power dynamics that have continued to influence response AGW discourse, as well as for fostering the reflexivity among researchers necessary to facilitate a meaningful focus on emancipatory ESD in the African context (O'Donoghue, 2007; Lotz-Sisitka et al., 2017).

The study draws upon an array of diverse data sources, encompassing pertinent official documents and policy statements, scholarly publications offering academic and theoretical insights into AGW and its ramifications for ESD, as well as news articles that mirror public perspectives and media coverage of AGW-related debates. Noteworthy examples of such official documents include the United Nations Sustainable Development Goals (SDGs), the United Nations Educational, Scientific and Cultural Organization (UNESCO), and reports from the Intergovernmental Panel on Climate Change (IPCC). The research endeavours to critically probe and distil meaningful insights from this multifaceted corpus of sources, which represent a number of key stances in the ongoing discourse on AGW, as well as the role of AIK and possible ESD strategies in the African context. Thus, this study primarily engages in a theoretical discussion, synthesizing insights from diverse scholarly sources to construct a comprehensive understanding of the topic at hand.

### 3.1 Theoretical framework

This research draws from a synthesis of two potentially complementary paradigms, namely, Transformative Learning Theories (TLT) and the Ubuntu philosophy, to underscore the contribution of African Indigenous Knowledge (AIK) in addressing and reshaping the challenges posed by Anthropogenic Global Warming (AGW). While Mezirow’s framework for transformational learning primarily centres on the individual, the integration with Ubuntu in this context aims to shift the focus towards the “we-paradigm” that characterizes the African worldview (Seehawer et al., 2022). This study takes its point of departure in a broad acknowledgement that Indigenous communities around the world offer valuable insights into humanity’s relationship with the environment, all of which need to be brought into respectful dialogue to transform environmental-related challenges. As in traditional African communities, many indigenous communities acknowledge their coexistence with spirits and other species in their landscapes, viewing them as integral to their subsistence (Brightman and Lewis, 2017). This perspective challenges the separation of human culture from nature, which characterizes the Anthropocene’s alienation of humans from their environments.

Transformative Learning Theory (TLT), when coupled with Ubuntu and other indigenous worldviews, accentuates the capacity for introspection and critical dialogue, offering the potential to engender profound shifts in individuals’ values and perspectives, particularly regarding their relationships with the environment (Diduck et al., 2012). TLT underscores the significance of third-order learning, a profound restructuring of fundamental beliefs, emotions, and behaviours, resulting in a shift in consciousness and a transformation of one’s way of engaging with the world. Additionally, TLT underscores the imperative for epistemological and perceptual evolution, fostering a transpersonal ethical orientation and participatory sensibility (Seehawer et al., 2022; O'Sullivan et al., 2016). In applying the TLT within the framework of AIK, this research endeavours to provide a more meaningful and contextually relevant response to the discourse surrounding AGW. This approach is designed to counter the continued marginalization of indigenous perspectives within the African educational system, even amid ongoing efforts to decolonize education, resulting from colonialism and Westernization (Ndlovu-Gatsheni, 2015). In this particular context, decoloniality, functioning as both a critical intellectual theory and a political project, aspires to disentangle formerly colonized regions from the persisting impacts of coloniality across its multifaceted manifestations (Smith et al., 2018; Opoku and James, 2021). This endeavour holds the potential to enhance the socio-cultural resonance and relevance of climate change education within the African milieu, where indigenous knowledge has often been inadequately integrated into educational curricula and processes (Risiro, 2019; McKinley, 2019).

Hence, an Ubuntu-inspired transformative learning approach serves to emphasize the frequently overlooked significance of African-inspired contributions in discussions surrounding responsibility and responsiveness to ESD. This helps to underscore the capability for self-reflexivity and engagement in critical discourse, with culturally relevant inputs from an African perspective (Seehawer et al., 2022; Diduck et al., 2012; Mezirow, 2003). These capabilities have transformative potentials on values and perspectives through which the individual worldviews including the relationship with the environment are shaped (Diduck et al., 2012). Such transformation is arguably beneficial, in terms of ethical and epistemological approach, toward the environment, with implications for AGW and climate change challenges (Braun et al., 2018). For instance, TLT highlights the presence of meaning structures that are shaped by specific beliefs, attitudes, and emotional responses, which are deeply influenced by one’s context and cultural background. These factors play a critical role in determining how individuals engage with and behave towards their environment (Romina, 2014).

Advocates of the TLT emphasize the significance of “third-order learning” or “epistemic learning” due to its potential to bring about a profound shift in one’s worldview. Third-order learning represents a transformative process that involves a fundamental reconfiguration of beliefs, emotions, and behaviours, resulting in a lasting and substantial transformation in how individuals perceive themselves, their position in the world, and their interactions with both other people and the natural world (Lange, 2019; O'Sullivan et al., 2016). This accents on “epistemological and perceptual change, and a transpersonal ethical and participative sensibility” (Loeber et al., 2007, p. 72) provide insights on how to build the necessary response to the AGW discourse, especially if applied within an African indigenous knowledge worldview (Tanyanyiwa, 2019). Hence, this study highlights the profound intricacies, implicit biases, and power dynamics that underlie the discourse on AGW, particularly in its implications for ESD within the African context.

## 4 Discussions: African indigenous transformation of AGW

Latour (2017) has asserted that climate change is the ‘revenge of Gaia’ against the modern view that strips nature of agency and reduces it to an object for human manipulation. Latour argues that to escape this modern trap, we must rethink our relationship with the environment and consider alternative cosmological standpoints, such as those held by Indigenous communities. Conversi (2021, p. 3) has also underlined that more attention to such “social organisations and human communities that are best qualified to act as ethical signposts and reference points for the preservation of life on Earth and the protection of future generations,” with the view to learn from them and transform our response to environmental challenges. The Ubuntu worldview, which espouses core principles of reciprocity, harmony, and solidarity among Indigenous societies within and beyond Africa, provides a transformative pathway to rethink and respond to the climate change crises.

The TLT framework, as explored in this study through the Ubuntu perspective, aims to accent deeper reflection on both our interrelatedness with all beings but also our ethical responsibility toward these beings (Ramose, 2015; Seehawer et al., 2022), reduce the tendency to view nature merely as an object, which has been a significant contributor to environmental degradation, and encourage active engagement with AGW. In its pursuit of ESD, this framework’s focus on human dignity and equality is valuable in rejuvenating a respectful exchange of knowledge that has often been overshadowed by Western intellectual dominance (Seehawer et al., 2022).

The incorporation of African Indigenous Knowledge (AIK) can reshape the objectives of UNESCO’s ESD by highlighting the essential role of culture. In the face of the influence of scientism in the African education system, there is a prevalent misunderstanding of AIK, with a tendency to validate Indigenous knowledge only through conventional scientific methods (Matolino and Kwindingwi, 2013; Shava, 2013). Such an approach restricts its applicability and effectiveness in the AGW discourse often framed in the technical language of Western science, neglecting values and spirituality central to ethical responsibility toward environmental sustainability (Okoliko, 2018; Braun et al., 2018). Thus, adopting a decolonial approach to AGW, informed by AIK, especially when rooted in Ubuntu, is justifiable both on epistemological and ethical grounds if the pursuit of climate change action is to be contextually relevant within the African education system. For instance, Ubuntu, as an example of epistemic and ethical worldviews, along with other related Traditional Ecological Knowledge (TEK), can offer an African-inspired perspective to ESD. This approach acknowledges the existence of other exemplary ethical communities (EEC), defined as “human communities with a proven track record of sustainability related to forms of traditional knowledge and the capacity to survive, sometimes thrive, outside the capitalist market and the nation-state system” (Conversi, 2021, p. 1). Now, let us further engage how Ubuntu, particularly in conjunction with the EEC model, can serve as a source of inspiration for ESD.

### 4.1 Ubuntu and sustainable development

The Ubuntu worldview, as expressed in its diverse forms, fundamentally embodies the concept of ‘humanness’ in its profound sense, deeply rooted in various cultures throughout sub-Saharan Africa (Ramose, 2015; Okoliko and David, 2021). This idea is articulated through linguistic variations such as *umundu* in Kikuyu (Kenya), *bumuntu* in kiSukuma (Tanzania), *gimuntu* in kiKongo (DRC), *botho* in Sotho (South Africa), *hunhu* in Shona (Zimbabwe), and more (Kamwangamalu, 1999). These variations share common organizing principles, including a sense of ‘groupness,’ unity, and shared identity, emphasizing the interconnectedness and mutual reliance of all beings (Samuel and Fayemi, 2019). Ubuntu, frequently encapsulated in the phrase “I am because we are,” underscores the idea that individuals are intrinsically linked within a communal context (Mbiti, 1990, p. 106). This view of the fundamental connections between individuals, communities and the environment reflects core values that are widespread across the African continent. References to sayings such as “It takes a village to raise a child” underscore the concept of communal responsibility and the cultural interdependence that characterizes this moral worldview (Mugumbate and Nyanguru, 2013, p. 95).

This African perspective of ‘humanness’ emphasizes that “a person is not just a passive ‘exister’ or existent but one who consciously initiates actions of effect towards beings other than the self, and by this means enhances the self” (David, 2020, p. 172). Impliedly, this worldview sees human persons as responsible for the preservation of the universe since their existence is intrinsically bound up as a dependent part of the whole (universe) (Samuel and Fayemi, 2019). It also emphasizes the interdependence between human beings as well as between humans and the physical world (Seehawer et al., 2022). Ubuntu’s communitarian outlook situates human-environmental relationships and respect for human dignity within an overarching oneness of all beings or “one community of life” (Van Norren, 2022). Accordingly, communities that espouse Ubuntu would qualify as exemplary ethical communities due to Ubuntu’s profound emphasis on interconnectedness, promotion of compassion and solidarity community-centeredness, alignment with sustainability, human dignity, ethical decision-making, restorative justice, and respect for nature (Ramose, 2015; Murove, 2009; Van Norren, 2022). These virtues encourage individuals to recognize their inherent connection with others, fostering a deep respect for human dignity and a commitment to making ethical choices that benefit the community. For instance, Ubuntu’s focus on restorative justice prioritizes healing and reconciliation over punitive measures, reinforcing its dedication to the well-being of the community (Van Norren, 2022; Seehawer et al., 2022; Moyo, 2021a). The community-centred perspective encourages empathy, solidarity, and a shared sense of responsibility among its members, while its ecological awareness underscores the importance of sustainability and respectful coexistence with the natural world, making Ubuntu a powerful example of an ethical community with holistic values that resonate with ethical and sustainable living.

The Ubuntu worldview, while holding a central place in many communities across the continent, has sadly faced a systematic process of marginalization, devaluation, suppression, and relegation within the colonial formal education system in Africa over the years, despite its acknowledged merits and practical applications (Okoliko and David, 2021). This has led to critical debates about its continued relevance in modern African societies (Koenane and Olatunji, 2017; Matolino and Kwindingwi, 2013). However, in line with the objectives of TLT inspired by Ubuntu in this study, persistently marginalizing such a crucial worldview not only undermines the call for inclusivity and epistemological diversity necessary for ESD policymakers and academics but also perpetuates the suppression of African-inspired wisdom in shaping global agendas (Inusah, 2023). This, in turn, reinforces the existing power imbalances in global North–South relations across various domains by denying Africans the representation they need (Connell et al., 2017). Hence, a transformative approach to ESD, one that is culturally sensitive to the African realities would do well to find innovative ways of reviving the positive elements of Ubuntu, despite the challenges of adaptability to modern realities pointed out by Matolino and Kwindingwi (2013). The advocacy for its contribution to sustainability discourses in extant studies goes beyond presenting Ubuntu as merely a ‘narrative of return’ as purported by Matolino and Kwindingwi (2013). A positive disposition toward Ubuntu as an *ethic of becoming* would be to rather highlight the required attitude for building the necessary harmony among all beings (Koenane and Olatunji, 2017). Accordingly, Ubuntu’s spirituality has the potential to tackle the climate change challenges resulting from unethical technological progress. In the words of Steve Biko,

Western society seems to be very concerned with producing their technological know-how while losing out on their spiritual dimension. We believe that in the long run, the special contribution to the world by Africa will be in this field of human relationships. The great powers of the world may have done wonders in giving the world an industrial and military look, but the great gift has to come from Africa—giving the world a more human face (Biko, 1978, p. 46).

Although the Ubuntu philosophy has been somewhat subdued in modern-day African societies due to various internal and external factors and influences, it still offers a valuable contribution that warrants deeper engagement among scholars and policymakers, as reminiscent of its deployment in various disciplines (Seehawer et al., 2022; Moyo, 2021b). According to this worldview, everything, from human beings to the trees of the forest and the stones, is inhabited by spirits or life forces, often referred to as vital forces (Tempels, 1959). For example, traditional Africans not only acknowledge but also seek approval from the spirits inhabiting trees in the forest before cutting them down if necessary. This concept of the interconnectedness of all things, inherent in Ubuntu, rejects egocentric or anthropocentric attitudes that tend to drive AGW. Mkabela (2015, p. 287) argues that this interconnectedness must be understood “in a holistic manner; physically, socially, and spiritually. It also focuses on the development of the whole person; physical, mental, spiritual, and social.” Consequently, its significance in the global initiative to address universal challenges, such as climate change, arises from its emphasis on a universal interconnectedness that underpins the core ethical values present in all value systems. This includes a strong commitment to the well-being of all, encompassing both humans and the environment, and a commitment to maintaining integrity (Prozesky and Morove, 2009).

Senghor contends that African reasoning is intuitive and participatory, in which the subject and objects of observation, the natural and supernatural, the mundane and the divine, the material and spiritual, are all united in an inseparable oneness (cited in Murove, 2009). This perspective embodies a profound appreciation for living in harmony with the environment, as it reflects the belief that an individual’s well-being is intricately tied to their ability to coexist in unity with all elements of existence (Ramose, 2015). Nature is likened to a “spider web of which no strand can be made to vibrate without affecting the entire web” (Tempels, 1959, p. 61). If one part of the web is disturbed, every other part feels the consequences. Therefore, the preservation of humanity strongly relies on the preservation of the planet. Attitudes that underestimate the role of any component of our global system undermine the principle of interconnectedness and interdependency that sustains nature. In agreement, Murove (2009) observes that the African concept of personal well-being is intricately linked to the preservation of the cosmos.

Recent scientific developments continue to transform our understanding of environmental relationships, resonating with Ubuntu’s principles of interconnectedness. For example, the discovery that most DNA in the human body is microbial emphasizes that humans are not isolated entities, but complex ecosystems intricately connected to the environment (Moore, 2017). This redefinition of the environment—from the molecular to the biome level—challenges reductionist worldviews that separate humans from nature. Similarly, natural sciences illustrate interdependence through ecosystems, where living components—microbes, plants, animals, and fungi—interact with non-living elements like climate and nutrients (Okoliko and David, 2021). The food chain and energy flow demonstrate this interconnectedness: plants convert solar energy into chemical energy, animals consume plants, and fungi decompose organic matter, returning nutrients to the soil in a continuous cycle of interdependence. This dynamic reinforces that human activities are inseparable from ecological systems, aligning with Ubuntu’s worldview that well-being emerges from interconnected relationships. This resonates with several key elements of ESD and TLT (Table 1).

**Table 1:** Key emphasis of Ubuntu, education for sustainable development (ESD), and transformative learning theory (TLT).

Key features	Ubuntu	ESD	TLT
Core philosophy	Interconnectedness, mutual care, and shared humanity—the principle of “I am because we are” (Mbiti, 1990: 106)	Integration of social, economic, and environmental dimensions is vital to sustainability (UNESCO, 2020a, 2020b).	Personal transformation through critical reflection, for worldview changes (Lange, 2019; Mezirow, 2003).
Perspective on Relationships	Highlights the interdependence of all Life forms	Relational thinking between people, society, and the environment is key to fostering sustainable lifestyles	Encourages interconnectedness through transformative experiences and learning (O'Sullivan et al., 2016)
Nature vs. Humanity	Rejects the duality between humans and nature—humans are part of a greater whole, responsible for nurturing all life (Ramose, 2002).	Acknowledges interdependence between humans and ecosystems to address environmental challenges (UNESCO, 2020a).	Encourages re-thinking human-nature relationships through reflection on environmental assumptions (Mezirow, 2003).
Epistemology and focus of learning	Values Indigenous knowledge systems and collective wisdom over individualism (Koenane and Olatunji, 2017).	Seeks to integrate Indigenous knowledge and alternative epistemologies into curricula (UNESCO, 2020a).	Focuses on challenging and reconstructing assumptions to develop new ways of thinking.
Goal of development	Seeks to promote communal harmony, balance, and environmental care (Murove, 2009).	It aims to create sustainable communities and responsible citizens through education.	Empowers individuals to adopt new values and act toward social and environmental change
Values and ethics	Emphasis on ethics of care, compassion, and mutual respect for people and nature (Ramose, 2015).	Promotes ethical decision-making for sustainable living	Encourages self-awareness and value shifts through transformation.
Approach to change	Emphasizes collective responsibility and gradual shifts through community dialogue (Kyei-Nuamah and Peng, 2024).	Seeks systemic change through education, policy, and social participation (UNESCO, 2020a)	Focuses on individual transformation as a precursor to broader social change (Mezirow, 2003).

The above table indicates how Ubuntu, ESD, and TLT converge in fostering environmental sustainability by challenging dualistic thinking that separates humans from nature and promoting interconnectedness. Ubuntu’s ethical foundation emphasizes responsibility for the environment, aligning with ESD’s goal of sustainable practices through education. Meanwhile, Transformative Learning Theory helps foster the individual mindset shifts necessary for meaningful engagement with sustainability efforts. Together, these frameworks suggest that sustainable solutions require both collective and individual transformation, grounded in the understanding that people, ecosystems, and economies are interdependent. TLT complements both by encouraging critical reflection to reimagine human-nature relationships (O'Sullivan et al., 2016). This concept of interrelatedness presents a valid critique of the tendency to objectify nature as an entity to be thoroughly examined and comprehended with absolute certainty before any actions are undertaken towards its preservation. This study argues that recognizing these relationships is essential for fostering a sense of human responsibility and agency in promoting sustainability within the framework of Education for Sustainable Development (ESD). As the foregoing suggests, modern epistemologies, which often impose artificial boundaries between humans and nature, foster exploitative ideologies that treat the environment as a separate, disposable resource (Escobar, 2015). ESD however challenges these frameworks by promoting holistic thinking and mutual responsibility, encouraging learners to see beyond rigid categories and appreciate the relational dependencies that sustain life (UNESCO, 2020a). As scientific and Indigenous perspectives converge on the idea of interdependence, it becomes evident that fostering sustainable behaviours requires a shift towards relational epistemologies—whether through Ubuntu or modern science—that embrace interconnectedness and shared responsibility.

### 4.2 Implication for climate response

African Indigenous Knowledge (AIK) represents a form of science deeply rooted in African culture and experiences, much like how Western science reflects Western culture and experiences. It is thus essential to recognize that the term “Western” primarily pertains to a specific approach to knowledge rather than the entirety of science itself. This understanding should pave the way for necessary democratizing the knowledge system in Africa, both in advocating for epistemic justice and in promoting an ethics-based approach to the natural environment. Ethical responsibility plays a pivotal role here and distinguishes between knowledge-driven care for the Earth and respect-driven care for the Earth. Without undermining the former, AIK aligns well with the latter, emphasizing the acknowledgement that individuals cannot exist independently of the environment, nor can they achieve absolute certainty in their knowledge. In contrast, a narrow focus on scientism in climate change policy tends to exacerbate rather than resolve the governance challenges associated with climate change due to the overemphasis on certainty as the basis for action.

By juxtaposing AGW-related dialogues with African Indigenous knowledge systems, such as Ubuntu, the research strives to shed light on its central tenet that ought to be integrated into educational curricula and programs. This endeavour is not only pivotal for fostering inclusivity in collectively addressing the challenges posed by AGW and climate change but also for advancing the discourse on ESD in Africa. Consequently, the ensuing discussion illustrates how this framework can contribute to the decolonization of ESD by addressing the challenges associated with scientism within the climate change discourse. This is where the demand for a profound transformation in the way knowledge and ethics are approached in African educational environments becomes apparent in addressing these challenges. The necessity for these adjustments becomes evident when we acknowledge the historical neglect of indigenous viewpoints within the colonial education system, especially in the context of the ‘Third World,’ with a specific emphasis on Africa (Mubangizi and Kaya, 2015). Despite the growing emphasis on decolonizing education in Africa, there still exists a notable absence or inadequate recognition of indigenous viewpoints, even among Africans themselves, thereby undermining its contribution to sustainable development (Lotz-Sisitka, 2004; Van Norren, 2022; Teffo, 2019).

A decolonial approach to education holds significant promise in rendering climate change education more socially and culturally meaningful and relevant within the African educational system, which has traditionally featured minimal indigenous content and processes (Mubangizi and Kaya, 2015). AIK’s emphasis on relationality, as observable in Ubuntu, discourages an obsession with quantifying the finer details at the expense of commitment toward ecological responsibility based on a deep sense of the interconnectedness of all beings (Okoliko, 2018). It sidesteps the hesitance toward actions advocated by denialists based on the inconclusiveness of climate science. It is true that even as more research continues to roll out evidence, grey areas remain in climate science, and we see this as a necessary part of science. This underscores the significance of embracing a positive stance towards democratizing knowledge, as advocated in the TLT inspired by Ubuntu (Seehawer et al., 2022). An Africa-inspired epistemic contribution to climate debates driven by denialist stances is to underscore that ethical responses to preserve the planet are necessary regardless of any uncertainties in climate projections or attribution.

The consensus position presented already in the 2013 IPCC report, that “human influence has *likely* been the dominant cause of the observed warming since the mid-20^th^ century could be perceived as still reserving a room for uncertainty regarding human culpability” (IPCC, 2013, p. 12). According to the IPCC confidence level evaluation, the term ‘likely’ stands for a confidence level of between 66 and 100%, which is indeed a strong confidence level. Wording in IPCC reports reflects difficulties in achieving consensus, considering both the large number of authors in these reports, and the involvement of state representatives in formulating final drafts. It also reflects a stance typical of scientific reporting, where limitations and uncertainties to any conclusions need to be explicitly stated. Nevertheless, the IPCC’s advocacy for action even amid uncertainty (IPCC, 2019) suggests an appreciation of the ethics of care required, which is very much consistent with what Ubuntu would advocate based on its intuitive appreciation of the principle of interrelatedness, which holds that all existence, including spirit beings, nature, the dead, the living, the unborn, etc., are intrinsically bounded in an interrelated and interdependent network (Murove, 2009; Samuel and Fayemi, 2019; Seehawer et al., 2022). Thus, an Ubuntu-inspired transformation highlights the contribution inherent in EEC as a response to contemporary environmental challenges.

The movement from agricultural to industrial society, from the divinely ordained certainties–uncertainties of the pre-modern age to the self-assured, proud, optimistic hubris of modernistic hegemony, is often conceived as a radical departure and watershed. It is customarily considered to be the greatest shift since the agricultural revolution. Therefore, those communities that continue practices dating back to previous generations (antecedent to the current global anthropogenic changes) can come to our help in various ways (Conversi, 2021, p. 4).

As Ramose (2015) observed, human beings are truly, an intrinsic part of nature although possibly a privileged part. Caring for the physical world is not merely a luxury but an imperative, as its neglect carries dire consequences on a global scale. In recognition of the interconnectedness of ecosystems and the shared fate of all living beings, the concept of “survival cosmopolitanism” has emerged as a pertinent perspective in environmental discourse (Conversi, 2020). Survival cosmopolitanism challenges traditional notions of nationalism by advocating for a more comprehensive and inclusive approach to addressing environmental challenges. This perspective underscores the shared responsibility that individuals and nations bear in ensuring the well-being of the planet. As highlighted by Posocco and Watson (2022), survival cosmopolitanism urges a shift from isolationist tendencies toward cooperative efforts on an international scale. It emphasizes the ethical imperative of considering the impact of individual and collective actions on a global level, promoting sustainability, and acknowledging the interdependence of human societies and the natural world. In adopting survival cosmopolitanism, environmental discussions aim to transcend geographical boundaries and prioritize the health of the planet for the benefit of all living beings, emphasizing a holistic and collaborative approach to safeguarding the Earth’s future. Herein lie the positive contribution of the we-paradigm of Ubuntu in transforming nationalism for the above goal.

Considering the discussion above, the necessary ethical response must also confront the self-serving strategies of nation-states, which undermine the call for a geoethical framework in climate change response. This is because the escalating ecological crises disregard the artificial borders set by states, highlighting their transborder nature and affirming the interconnectedness of all entities, including the nation-states themselves (Okoliko and David, 2021). Sometimes, the ethical perspectives of these nation-states run counter to the essential geoethical outlook required for an effective response to AGW. As aptly noted by Conversi, “From a geoethical standpoint, the Earth should be the primary unit of analysis and the guiding tool for comprehending and navigating the realities of a global crisis” (Conversi, 2021, p. 5). This underscores the necessity for international collaboration driven by the awareness of the intrinsic interdependence among nation-states (Conversi, 2021; Okoliko and David, 2021). Accordingly, the incorporation of Ubuntu’s we-paradigm into the discourse of survival cosmopolitanism provides a positive and transformative avenue for reorienting nationalism toward collaborative environmental goals. By fostering a sense of interconnectedness and shared responsibility, Ubuntu contributes to a more inclusive and ethical approach to addressing global environmental challenges, and in a manner consistent with the EEC orientation (Okoliko and David, 2021).

Thus, an epistemological shift on the AGW issues toward embracing an African-inspired perspective of holism is more desirable than adhering to a more limited viewpoint that jeopardizes the commitment to safeguarding the planet. To this end, Olatunji (2010, p. 19) averred that “the object-subject and the ‘we versus them’ outlook of demarcationist epistemology associated with rationalism and empiricism need to be replaced with a spontaneous and integrative holism.” This perspective places significant emphasis on Ubuntu’s recognition of symbiotic relationships between humans and the environment, particularly its implications for the conservation of the natural world.

Drawing on Ubuntu philosophy in ESD does not merely concern the content, values and learners’ understanding of self, but also has implications for which forms of knowledge transmission are considered valid. Thus, according to Le Grange (2012), Africans have found folklore as a means of conveying moral instructions. Within traditional African folktales, the natural world assumes a central position, frequently featuring characters beyond human beings. These narratives often portray situations where an animal rescues a vulnerable human. These tales are laden with moral depth as they demonstrate the active involvement of nature in human affairs. They serve to remind us that humans are not entirely self-sustaining, despite their apparent privileged status within the hierarchical order. By infusing such narratives of mutual harmony between humans and animals, a significant reorientation of human agents towards ethical and responsive interactions with nature can be achieved, in line with the perspective of an Ubuntu-inspired TLT. For instance, in certain African stories, human protagonists are ready to humble themselves to be saved by small animals that have befriended them (Fortune, 1974). On such folklore, Murove surmizes that the way small animals like tortoises can outsmart big ones like lions and elephants emphasizes how virtues such as humility, compassion, and solidarity, which are the basis of the notion of *Ukama*, are shown to exist in both *muntu* and the natural environment (Murove, 2009). By implication, this parallel shows the interdependence that exists between the two, the consciousness of which enables the *muntu* to draw the lesson from the natural world with greater flexibility and according to the moral requirements of the occasions.

### 4.3 Towards decolonized education for sustainable development

An obsession with getting the science right on the human-earth links vis-a-vis global warming before climate action has no basis in the indigenous worldview. The subordination of the African way of life within the education system has however restricted the potential contribution of African Indigenous knowledge in the AGW discourse. Hence, the thrust of the decolonial approach through the TLT espoused in this study involves promoting the interdependence and integration of diverse forms of knowledge and cultures in the drive toward ESD with the view to transform the means of achieving this end. The dominant orientation of the sustainable development Goals (SDGs) has been appositely criticised for its relative silence on the centrality of culture in development (Boogaard and van Norren, 2021). It conceives development as merely “an autonomous process whose end-product is delivered to people” (Ntibagirirwa, 2012, p. 218). This so-called ‘extroversion’ makes development merely an “expansion of people’s capability,” which partly explains why ‘development’ as advocated within the education system influenced by colonialism often inadequately incorporates African values and spirituality, as these cannot easily be reduced to products. Extroversion so construed, consists of a lack of appreciation for one’s values, beliefs, and potentialities due to the adoption of a foreign values system. This has been understood as part of the colonial legacies that undermine Africans’ right to self-determination; hence, the relevance of decoloniality in seeking to transform this reality by reintroducing these values.

The backwards-looking and corrective sense of decoloniality “is fundamentally a normative principle grounded in human equality and respect for human dignity” (Manthalu and Waghid, 2019). Such respect for human equality and dignity is still lacking in climate change discourse and governance as relevant bodies such as the IPCC and the academic community continue to privilege Western voices despite admitting the relevance of Indigenous and local knowledge. The recent World ranking of 1,000 top climate scientists in the so-called Reuters Hotlist is reminiscent of such biases against indigenous voices (Hunter et al., 2022: n p), and has generated some debate on the continued delegitimization of indigenous voices through subtle under-representation of non-western worldview. Hunter et al. (2022) observed that “While over three-quarters of the global population lives in Asia and Africa, over three-quarters of the scientists on the list are located in Europe and North America. Only five are listed for Africa.” The criteria that underpin the noticeably skewed list in favour of scientists from the Global North reveal not only the inequality produced by various metric-based validations of what counts as climate science but also bring to the fore the politics of knowledge production that privileges the West over the rest. It suggests an underappreciation of the positive influence of “local researchers, drawing on contextualized and decolonized global knowledge,” on policymakers and practitioners affecting climate change solutions, for instance, in the context of South Africa (Hunter et al., 2022). The decolonized and contextually rooted contributions play a significant role in advancing the essential goal of epistemic democratization, particularly in promoting the African worldview within the discourse on AGW (Hunter et al., 2022).

While advocating for an understanding of the African perspective, this normative principle of decoloniality also possesses a “forward-looking” dimension as it challenges any uncritical form of essentialism in the sense of decoloniality that looks backwards and aims to correct historical imbalances. This normative dimension avoids the tendency to equate “decoloniality with an Africanization” in a manner that excludes other perspectives (Manthalu and Waghid, 2019). Maintaining human equality and respect for human dignity is hardly feasible while simultaneously marginalizing the African perspective in education at large, including its contribution to ESD discourses. Within the African educational context, decoloniality necessitates an “intellectual and ultimately structural re-examination of the enduring and self-perpetuating harmful imperialist histories of the world today” (Ndlovu-Gatsheni, 2015, p. 485).

Scholars have drawn upon the wealth of insights from the African philosophy of education to assess the general approach to knowledge production and dissemination in African education, including higher education (Msila, 2009; Etieyibo, 2017). Recognizing the myriad challenges in African universities, these scholars rightly argue for an expansion of epistemology that bridges the gap between Western and African philosophies, addressing their respective shortcomings. This approach aligns with the idea of ‘universal knowledge systems,’ understood as a knowledge system that focuses on the unity of knowledge rather than the promotion of one form of knowledge over another (Moll, 2002, p. 10). The required epistemic democratization is one that critically deconstructs both the suppression of local or Indigenous knowledge and the universalization of Western knowledge in education systems across Africa.

Accordingly, we emphasize some of the elements of Ubuntu, which qualify it as an EEC because it provides a valuable model for ethical living and community well-being. In acquiescence with Seehawer et al. (2022), I argue in this study that turning to the principles of Ubuntu can be considered a transformative approach to sustainability education within the EEC model. Conversi’s observation regarding the need for recourse to ECC suggests that the above principles “can no longer be seen as anachronisms or as utopian and romantic ideals,” especially considering the limitations of Westernization (Conversi, 2021, p. 4). These limitations encompass the recognition, as highlighted by Zeng et al. (2020), of the insufficiency of the United Nations’ sustainable development goals (SDGs) in safeguarding the Earth. Indeed, “In these liminal times, the Earth needs to be reconceived as a plural-cultural space in which distinct types of human communities engage in different levels of ethical responsibility to allow the survival of life by transferring Earth-system knowledge to coming generations” (Conversi, 2021, p. 1). To this end, Conversi argued that “the protection and maintenance of these EECs can become the crucible in the struggle for the survival of humankind and other forms of life” (2021, p. 1).

In the African context, the rapid erosion of Indigenous values, particularly in the domains of epistemology and ethics, due to colonialism, underscores the need for more effective approaches to the decolonization efforts needed to preserve Indigenous values and spirituality. Hence, the forward-looking perspective of decoloniality provides the necessary space and a point of departure for the transformational learning theory concerning the goal. It promotes a level of responsiveness on the part of the agent of education/learning toward the AGW discourse in a social and culturally relevant manner. Advocating for the need for a healthy hybridity of perspectives, Manthalu and Waghid (2019, p. x) argue that “ideal decoloniality calls for a critical study of all perspectives as legitimate equal objects of knowledge without unduly privileging and prejudicing some perspectives.”

The leadership and policymakers in African education systems must foster the requisite transformation that will make ESD more relevant and responsive to the demands of climate change mitigation and adaptation. The achievement of such a goal demands concerted effort in the Africanization of the processes and outcomes of education. In the context of transformative learning theory and its relationship with Ubuntu, the aim is to render climate change education culturally meaningful for Indigenous and local learners. Achieving this objective demands not only a deeper understanding of the African Indigenous perspective on ESD but also a critical evaluation of the current position of this perspective within the educational system. The goal is to emphasize the necessity for individuals to reconnect with their cultural roots. I contend that this is crucial for shedding light on the subtle influence of scientism in Africa’s higher education has created a barrier to the flourishing of Indigenous wisdom, which, in turn, may hinder culturally informed responses to global warming and climate change. In this context, Ubuntu-inspired transformative learning primarily seeks to empower African agencies, given the historical suppression of such agencies in mainstream educational settings. It aims to re-establish a sense of autonomy and cultural identity while simultaneously addressing global environmental challenges through a perspective that respects and draws from Indigenous wisdom and ethical principles, both within and beyond Africa.

Over the years, Eurocentric epistemological dominance in education has led to a devaluation of local and Indigenous knowledge to the extent that a concerted effort is required to revive Indigenous knowledge systems. This revival is transformative in that it intends to raise awareness among Africans of their right to balanced epistemological representation and their responsibility to bring about this reality through their agency. This calls for transformative action to combat climate change, highlighting the importance of reshaping sustainability discussions with a localized or Indigenous focus while drawing applicable insights from the EEC model.

In the realm of ESD, an Ubuntu-informed transformative learning approach offers valuable perspectives for this necessary transformation. It draws inspiration from Ubuntu and other Indigenous knowledge systems that align with the EEC framework. In situations where these valuable contributions to ESD have been marginalized, as is often the case in African education systems, “transformation and decoloniality involve re-centring perspectives, experiences, and epistemologies that have been unfairly marginalized in academic spaces” (Manthalu and Waghid, 2019, p. 66). This re-centring is critical for fostering a comprehensive understanding of decoloniality, one that acknowledges and prioritizes the significance of Africa’s Indigenous Knowledge (AIK) in addressing AGW and its consequences.

## 5 Conclusion

The profound repercussions of climate change on both humanity and the broader ecological community emphasize the urgent need for a shift in attitudes toward environmental sustainability. This necessary shift, however, has been slow to materialize, largely due to the predominant reliance on a ‘fact-driven-only’ approach championed by proponents of scientism within the discourse of AGW. This situation highlights a compelling argument against placing exclusive reliance on science-based facts as the sole foundation for our ethical commitments to ESD. In this study, I attempted to illustrate that scientism not only contains inherent self-contradictions, as its claims lack a scientific basis, but it also leads to ethically misguided responses to climate issues among its adherents—many of whom are not necessarily scientists themselves.

An approach to ESD that fully embraces Indigenous wisdom alongside Western science has the potential to address the shortcomings of scientism in sustainability education. It cultivates a mindset enriched with values that hold the environment in high regard, a crucial aspect of genuinely decolonized sustainability education in Africa. This approach recognizes that our understanding of ecosystems ultimately shapes how we interact with the elements within them.

Hence, the response to AGW within mainstream education should not be exclusively grounded in the Western epistemic paradigm. Instead, it should incorporate Indigenous wisdom, which places paramount importance on the interconnectedness that exists among all elements of existence, including epistemic interconnectedness. This perspective consistently reminds us of the limitations of various forms of knowledge and the significance of showing reverence in our relationship with the environment. The approach aligns with the perspectives offered by Ubuntu-inspired transformative that incorporates spiritual or transcendental beliefs into African policymaking to address climate problems in its education system, potentially providing a balanced response to the climate change crisis by avoiding the extremes of denialism and ecomodernism. This entails not only the expansion of our knowledge boundaries but also acknowledges the essential role of African agency in this expansion, considering Ubuntu’s emphasis on responsibility towards one another. This agency is also relevant in the imperative to integrate AIK into the mainstream education system in Africa, thereby enhancing the effectiveness of sustainability education.

## Data Availability

The original contributions presented in the study are included in the article/supplementary material, further inquiries can be directed to the corresponding author/s.
